# Wrestling with a ghost: facing an opponent I can neither see nor clinch – the experience of professional wrestlers who have suffered an ACL injury

**DOI:** 10.1136/bmjsem-2023-001782

**Published:** 2024-03-12

**Authors:** Ramana Piussi, Elin Nilsson, Hannah Karlsson, Martin Hägglund, Andreas Ivarsson, Kristian Samuelsson, Hans-Christer Holmberg, Eric Hamrin Senorski

**Affiliations:** 1 Sahlgrenska Sports Medicine Center, Gothenburg, Sweden; 2 Health and Rehabilitation, Institute of Neuroscience and Physiology, Sahlgrenska Academy, University of Gothenburg, Gothenburg, Sweden; 3 Lund, Sweden; 4 Division of Physiotherapy, Department of Medical and Health Sciences, Linköping University, Linköping, Sweden; 5 Sport Without Injury ProgrammE (SWIPE), Department of Health, Medicine and Caring Sciences, Linköping University, Linköping, Sweden; 6 Swedish Olympic Committee, Stockholm, Sweden; 7 School of Health and Welfare, Halmstad University, Halmstad, Sweden; 8 Department of Sport Science and Physical Education, University of Agder, Kristianstad, Norway; 9 Department of Orthopaedics, Institute of Clinical Sciences, Gothenburg, Sweden; 10 Department of Health Sciences, Luleå University of Technology, Luleå, Sweden; 11 Department of Physiology and Pharmacology, Biomedicum C5, Karolinska Institutet, Stockholm, Sweden

**Keywords:** Knee, Sports medicine, Qualitative Research

## Abstract

This study explored professional wrestlers’ experiences of the consequences of an anterior cruciate ligament (ACL) injury and their perception of whether the ACL injury could have been prevented. We interviewed 10 professional wrestlers (60% women, age range 21–34) treated with ACL reconstruction with semistructured interviews. Transcripts were analysed using qualitative content analysis: One major theme, ‘Wrestling with a ghost: facing an opponent I can neither see nor clinch’, supported by five main categories, emerged from the collected data. The five main categories were: My ACL injury: bad luck or bad planning?; The way back: a fight to return to sport; Only performance counts; The injury’s impact on life: a wrestling with emotions; In hindsight, personal growth. Professional wrestlers who experienced an ACL injury expressed that not only the injury itself but also the subsequent recovery posed major challenges that they did not know how to deal with and that, in some cases, ended the athletes’ wrestling careers. Professional wrestlers attributed their ACL injuries to bad luck or large training loads and wished that they had more support from the wrestling community when injured.

**WHAT IS ALREADY KNOWN ON THIS TOPIC**

- Professional athletes who suffer an anterior cruciate ligament (ACL) injury go through life-changing processes and do not always return to perform as before the injury.

**WHAT THIS STUDY ADDS**

- Professional wrestlers who suffered an ACL injury experienced both the injury itself and the recovery process as highly challenging. They reported a lack of sufficient support from the wrestling federation, and, in half the cases examined in this study, the injured ACL forced the athletes to abandon their professional careers. Some professional wrestlers thought a long, intensive training period without recovery caused their injury.

**HOW THIS STUDY MIGHT AFFECT RESEARCH, PRACTICE OR POLICY**

- Trainers and healthcare providers working with professional wrestlers should acknowledge that the rehabilitation after ACL reconstruction is very physically and mentally demanding and should provide every possible support by checking the wrestler’s perceptions of where support is needed. Further, trainers and healthcare providers involved in the rehabilitation of professional wrestlers can use this study to pass on the message that ‘this too shall pass’, and despite all the negative emotions perceived during rehabilitation, some positive lessons can be learnt.

## Introduction

 Wrestling is one of the most ancient sports in the world and was part of the old Olympic Games when they started in 776 B.C.^1^ Due to the extreme physical demands, the rates of injury for professional wrestlers are as high as 19.6/1000 exposure hours,^2^,^4^ which can be compared with 8.1/1000 hours in professional male football or 21.6/1000 hours in professional male American football.^5^ At the 2008 Beijing Olympic Games, the incidence of injury among professional wrestlers was 9.3/100 athletes.^6^ Across three consecutive Olympic Games (Beijing 2008, London 2012 and Rio de Janeiro 2016), the injury incidence in wrestling was lower (4.8 injuries/1000 exposure minutes) compared with judo (9.6) or taekwondo (7.7).^7^ However, wrestling had the highest proportion of injuries resulting in ≥7 days absence from practice (39.6%), compared with judo (35.9%) or taekwondo (32.5%).^7^ In professional wrestling, the second most common injury type (after skin abrasions) is knee injuries, including anterior cruciate ligament (ACL) injuries.^4 8^ An ACL injury in wrestling most commonly occurs when attempting a takedown manoeuvre with the weight-bearing foot stuck on the mat. The reported proportion of ACL injuries in wrestling ranges from 0% to 10% of knee injuries.^2 9^

An ACL injury can have devastating consequences for a professional athlete, for example, time loss, physical limitations, pain and fear, and can result in the end of a wrestling career.^8 10 11^ An ACL injury in professional athletes is commonly treated with surgical reconstruction and subsequent rehabilitation.^12^ In the aftermath of an ACL injury, the athlete will experience various situations associated with various emotions. Certain emotions, such as inspiration after successful interactions with others, are considered to facilitate the rehabilitation process, while others, such as kinesiophobia, appear to be barriers to the rehabilitation process.^13^ Athletes have been reported to struggle with expectations of full recovery,^14^ fear of reinjury and inferior perceived knee function, as well as inferior confidence and emotions related to returning to their sport, together with pressure to play even if they were not physically or psychologically fit.^15 16^ Professional athletes have been reported to suffer from loss of athletic identity after ACL injury^17^ and 6–12 months after ACL reconstruction.^18^ Up to 5 years after ACL reconstruction, interviewed patients might experience full symptom resolution or persistent symptoms and need to accept negative life-changing consequences due to the injury.^19^

Taken together, professional athletes who suffer an ACL injury go through a life-changing process. It is unknown how professional wrestlers experience the challenge of a life-changing process such as an ACL injury. To help professional wrestlers cope with the demands of this life-changing process, healthcare providers need insight into professional wrestlers’ experiences of suffering an ACL injury and the recovery process. As a result, professional wrestlers’ experiences of the consequences of an ACL injury and rehabilitation process are an important piece in the puzzle relating to returning professional wrestlers to perform on the mat. An ACL injury and the subsequent treatment are experienced differently by each individual. Therefore, qualitative studies that allow the patient’s voice to be heard by the research and clinical communities are highly warranted.

The present study was designed to explore professional wrestlers’ experiences of the consequences of an ACL injury and their perception of whether the risk of sustaining this injury could have been reduced.

## Method

### Study design and participants

We believe the experience of a serious knee injury is constructed differently by every unique individual. Therefore, in this qualitative interview study, we adopted an interpretive/constructivist epistemological inductive approach.^20^ More specifically, an injury and rehabilitation process is a highly individual experience. Respondents might be more prone to provide rich material in an individual interview setting, so individual interviews were chosen.^21^ The Consolidated criteria for Reporting Qualitative research (COREQ)^22^ checklist was used to report methodological information transparently.

An interview guide^23^ was developed through discussions between study authors. The interview guide was pilot tested with a former professional wrestler who suffered three ACL injuries and was not included in the analysis (author EN). No amendments were made to the interview guide after the pilot test. The interview guide was used as a template, and follow-up questions such as ‘Can you develop this further’ or ‘What do you mean by…’ were used when deemed appropriate. Box 1 presents the interview guide.

Box 1 **Interview guide**

How long have you been wrestling?

What does wrestling mean to you?

What is your goal with wrestling?

How and when did you injure your ACL?

What did you feel physically when you injured your ACL?

What did you feel psychologically when you injured your ACL?

What did you feel when you received the diagnosis?

What happened after the injury?

What kind of help did you receive after the injury?

Why do you think you injured your ACL?

Was there anything you could have done to avoid the injury?

How has this injury affected you as an athlete?

Could you tell me if the rehabilitation after the injury could have been organised differently?

How long were you in the recovery process?

What do you feel about your knee?

How does your knee affect you as an athlete?

How does your knee affect your performance as an athlete?

Would you have done anything differently if you could go back in time? In that case, what?

Is there anything you would like to add?

Do you have any suggestions for improving the ACL injury management and recovery process?

ACL, anterior cruciate ligament

A purposive sampling was adopted, where researchers intentionally select participants with specific characteristics or unique experiences related to the research question. The goal of purposive sampling is to identify and recruit participants who can provide rich and diverse data to enhance the research findings.^24^ Registered wrestlers who had participated at the elite level during the last 5 years and had suffered an ACL injury were identified by the Swedish Wrestling Federation, and contacted by the second author (EN) about participation in this study. Elite level was defined as competing at the highest international level, such as the European or World Championships. A maximum of 5 years before inclusion in the study was set to diminish the risk of recall bias and ensure the inclusion of recently active athletes within the sport. Athletes were informed about the study and asked whether they were interested in participating. Upon positive response, an interview was scheduled. Every interview was transcribed and analysed before the next interview was performed. Interviews were scheduled and performed until no further data (no new sub-categories) emerged from the analysis. All the athletes were informed that participation in the study was voluntary and that withdrawal from participation was possible without any explanation. All the statements from athletes were analysed confidentially. Oral recorded consent was collected. Ten athletes who respected inclusion criteria (six females and four males) were identified and contacted, and no one declined participation. Consequently, 10 individual semistructured interviews were performed. No relationship was present between the authors of the study and included athletes. Table 2 shows the demographics of the included wrestlers. Demographics were presented at the group level to ensure confidentiality.

**Table 1:** Demographics of the professional wrestlers included

Number	Age range	Years as a wrestler	Time between surgery and interview (months)	Had to give up competitive wrestling due to ACL injury
10 (60% W)	21–34	10–25	1–240	5 (50%)

ACLanterior cruciate ligamentWwomen

### Patients and public involvement statement

One of the authors (EN) is a former professional wrestler who has had three ACL injuries and provided clinical and patient perspectives in the conception of the study and the development of the interview guide. Neither patients nor the public were involved in recruitment to or the conduct of the study or were asked to assess the burden of participating in the study.

### Data collection

Data were collected during the autumn of 2022. Interviews were performed via the Zoom web-based application (Zoom Video Communications,San José, California, USA). All the interviews were conducted by the first author (RP). During the interviews, no field notes were taken. Interviews were recorded via the Zoom recording function. The mean interview duration was 26 min, ranging from 17 to 48 min.

### Data analysis

Qualitative content analysis was chosen to analyse the collected data since it is a structured qualitative analysis method that can be used within the constructivist paradigm.^25^ A further reason to adopt qualitative content analysis is that it is a flexible method for exploring latent and manifest content. The first author (RP) was primarily responsible for the data analysis process. Still, data were continuously triangulated^26^ among other authors (EN, HK, AI, MH, EHS) who checked the coding and categorisation process and where presumptions were continuously discussed. An extensive discussion took place between the authors involved in the analysis to increase the trustworthiness of the results. The analysis process is iterative and was performed back and forth through discussions between the authors involved. Transcripts were not sent to participants for corrections or comments.

The data were analysed using qualitative content analysis based on Graneheim and Lundman,^27 28^ according to the following steps:

- Transcripts were first read thoroughly to obtain a general understanding of the collected data by all authors involved in the analysis process (RP, EN, HK, EHS).
- Meaningful units were identified, extracted and shortened to condensed meaningful units. Condensed meaningful units were then abstracted in a Microsoft Excel spreadsheet. This step was performed by RP, EN and HK separately.
- Condensed meaning units were coded. This step was performed by RP, EN and HK separately.
- Codes were grouped for similarities and differences in subcategories. While grouping codes in subcategories, transcripts were read again several times and subcategories were validated against the transcripts to ensure that data were not missed or erroneously included. Grouping codes into subcategories was performed by continuous discussion between RP, EN and HK until a consensus was reached. Up to this stage, the authors made a strong effort to minimise interpretation and stay close to the text.
- Subcategories were grouped for similarities and differences into main categories. Grouping subcategories into main categories was performed by continuous discussion between RP, EN, HK and EHS until a consensus was reached. At this stage, a minimal interpretation of the content of the text was allowed.
- All authors involved in the analysis had extensive discussions to elaborate on the ‘professional wrestlers’ experiences of consequences of an ACL injury’. Interpretation of content at this stage was allowed, and finally, consensus over a theme was reached.

By analysing one interview before moving to the next one, a judgement of no new data emerging from the analysis can be made, and authors can reflect on their biases and assumptions while performing the data collection process. Any disagreement between the authors was resolved by discussion with the senior author.

With regard to transparency and reflexivity within qualitative research, the first (RP), the fourth (MH) and the senior author (EHS) are experienced male physiotherapists working in a sports rehabilitation and research setting. The first author is a PhD student, while the fourth (MH) and senior (EHS) authors are professors and associate professors. The second (EN) and third (HK) authors are two female physiotherapists with a special interest in ACL injury and rehabilitation. Author EN is a former professional wrestler who suffered three ACL injuries. Regarding the other authors, KS is an orthopaedic surgeon (professor) and a former elite judoka who has suffered an ACL injury on both knees. Author AI is a male researcher and sports psychology consultant (professor) with extensive experience in the research field. Author HCH is a male professor with extensive experience in the research field and, at the time of the study, was involved in the Swedish Olympic Committee. The first, sixth and senior authors have collaborated on several qualitative investigations. Authors MH, AI and HCH have extensive experience conducting qualitative research, while this was the first qualitative study for the second and the third author.

To increase qualitative research transparency, table 2 presents an example of the analysis process involving codes and the grouping into subcategories and main categories.

**Table 2:** Example of the analysis process from codes to main category

Codes	Subcategory	Main category
Most important in my life	Mental challenges during rehabilitation	The injury′s impact on life: a wrestling with emotions
Wrestling gives me meaning		
Guilt		
Wrestling is like a family		
I don’t trust my knee	Necessary adaptations due to the injury	
Accepted chronic pain		
Forced to lower my ambition		
Adapt everyday life		
Unfortunate	Influencing factors outside my control	My ACL injury: bad luck or bad planning?
Usual situation		
Hard to avoid this situation		
You cannot change what the opponent does		
Too little recovery	Influencing factors, I could have controlled.	
Much training just before a major competition		
I was under great pressure		
Unfocused (tired)		

## Results

One main theme, ‘Wrestling with a ghost: facing an opponent I can neither see nor clinch’, emerged from the collected data, supported by 5 main categories and 16 subcategories (table 3).

**Table 3:** The main and subcategories on the theme of ‘Wrestling with a ghost: facing an opponent I can neither see nor clinch’

Main categories	Subcategories
My ACL injury: bad luck or bad planning?	The injury mechanism
	Influencing factors outside my control
	Influencing factors, I could have controlled
The way back: a fight to return to sport	Healthcare system
	Focus on recovery
	Loneliness
	Stress due to inactivity
	Knee symptoms
Only performance counts	No support from the federation or trainer
	A desire for a major change in the federation
The injury′s impact on life: a wrestling with emotions	Mental challenges during rehabilitation
	Necessary adaptations due to the injury
	The support of family and friends
In hindsight, personal growth	Lessons learnt
	Alternative considerations

The theme could be summarised as ‘*Wrestling with a ghost: facing an opponent I can neither see nor clinch*’. Some of our professional wrestlers regarded their ACL injury as bad luck, which can happen when training or wrestling. In contrast, others felt that the injury resulted from a period of very high intensity training when they were extremely tired, both physically and psychologically. In the latter cases, the wrestlers felt the ACL injury approaching like a ghost: something hovering on the horizon that they could not quite glimpse.

After their ACL injury, the professional wrestlers received rapid help from the healthcare system. However, during rehabilitation, they reported having to cope with feelings of stress from not being able to train regularly, pressure because they were not in such good physical shape as before the injury, loneliness, as well as physical symptoms such as pain, stiffness and clicking in the knee. The wrestlers said that they had to face a new opponent: one that could not be grappled physically, an opponent composed of physical and psychological limitations and adaptations distant from their professional life and fraught with debilitation and challenges. All in all, the professional wrestlers experienced their ACL injury and the subsequent period as a very tough match, not against a human body but rather against challenges they were unprepared for, in a way like wrestling a ghost.

### My ACL injury: bad luck or bad planning?

In their responses to the main category, ‘My ACL injury: bad luck or bad planning’, the professional wrestlers described how their ACL injury occurred. Some wrestlers believed it was due to bad luck, whereas others thought their injury resulted from long, intensive training without adequate recovery. Nearly all the injuries occurred at the end of a training session, often during a demanding period with much training and competition. While preparing for a major competition, some wrestlers reported fatigue, felt their bodies sagging and had difficulty focusing when training. Their ACL was injured in this kind of tiredness or lack of attention.

#### The injury mechanism

**Figure 1:**
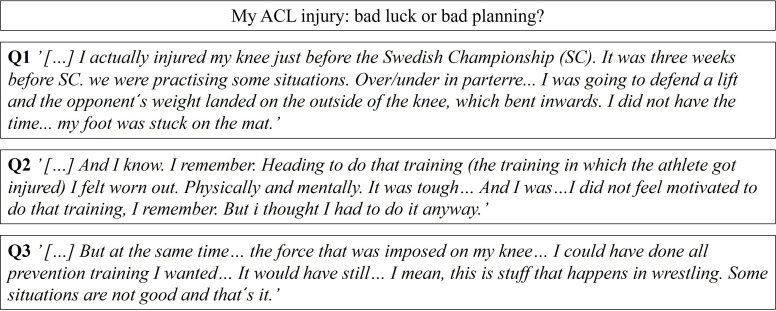
A selection of representative quotes relating to the subcategories in the first main category.

When wrestling, there is always an opponent to take into account. All the ACL injuries occurred during a defensive move or counterattack while wrestling. In all cases, the foot of the injured leg was on the mat, with the body rotated and the opponent who applied force on the upper body or the knee (Q1, figure 1).

#### Influencing factors outside my control

Professional wrestlers who reported that controllable risk factors preceded their ACL injury expressed that the injury was possibly preventable. Those professional wrestlers experienced a long, hard and demanding training period interrupted by the injury. They reported preparing for a major competition, for example, and not having the time or the opportunity to listen to their body (Q2, figure 1).

#### Influencing factors, I could have controlled

Some professional wrestlers reported that not everything on the mat can be controlled, for example, a foot gets stuck on the mat while the opponent pushes the knee into a valgus. Consequently, some of our wrestlers reported that the ACL injury was part of the sport and was unavoidable. Others said they were just unlucky and could not see the injury approaching in the period before the ACL injury (Q3, figure 1).

### The way back: a fight to return to sport

During rehabilitation, wrestlers felt stress from being away from training due to rehabilitation and tried to rehabilitate as quickly as possible. Interviewed subjects tried to ignore symptoms from the knee, such as pain or swelling, or ignored advice from physiotherapists not to return to the mat too early. A fighting attitude of simply getting through the demanding time represented by injury and rehabilitation was expressed. However, due to the fighting mentality, some subjects tended not to listen to their body and ignored knee symptoms generated by training too hard and too early. Then, returning to the mat too early, with the knee that gave symptoms such as pain or stiffness, led certain wrestlers to adapt their wrestling style to the physical limitations caused by the knee. In the whirlpool of feelings of stress and body symptoms, professional wrestlers experienced feelings of loneliness. The encounter with the healthcare system was perceived as positive, but more wrestling-specific knowledge by healthcare providers was desirable.

#### The healthcare system

**Figure 2:**
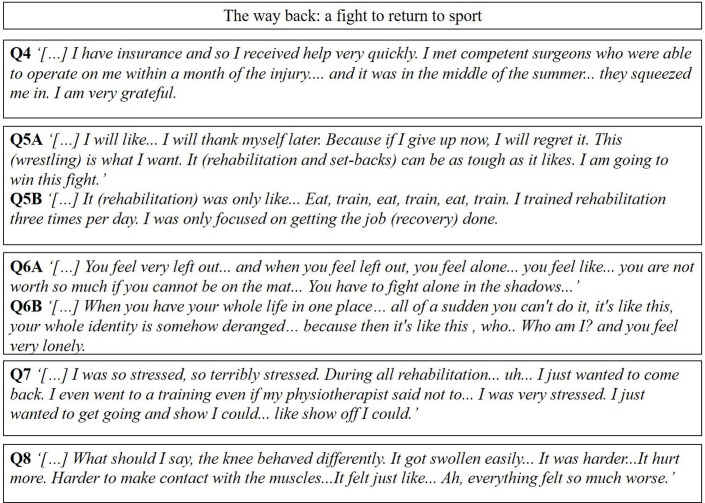
A selection of representative quotes relating to the subcategories in the second main category.

In connection with their positive encounter with the healthcare system, our subjects were satisfied with the process of diagnosis and surgery and the extensive support they received from physiotherapists during rehabilitation. However, some professional wrestlers had hoped for better wrestling knowledge from physiotherapists (Q4, figure 2).

#### Focus on recovery

As elite athletes, our wrestlers saw no other alternative than to fight back from the ACL injury to achieve their goal of being the best in the world. They reported wanting to get the job done to return to wrestling without looking back (Q5A and B, figure 2).

#### Loneliness

After the ACL injury, some of the professional wrestlers felt left outside the training group. Furthermore, not being able to participate in the activity that before the injury took up so much time made professional wrestlers feel unseen by the trainers, fighting all alone (Q6A and B, figure 2).

#### Stress due to inactivity

Professional wrestlers reported being stressed by missing time on the mat due to the ACL injury. The perceived stress was mentioned due to the missed opportunity to become a better wrestler and missing out on important competitions. Having the stress as a driver, our wrestlers reported wanting to get through rehabilitation as quickly as possible (Q7, figure 2).

#### Knee symptoms

During rehabilitation, the wrestlers experienced a variety of knee symptoms such as excessive pain, stiffness, knee joint effusion and reduced flexibility as a result of their ACL injury. Experiencing this variety of symptoms was perceived as rather negative by professional wrestlers in this study (Q8, figure 2).

### Only performance counts

According to professional wrestlers themselves, they still train as they did 60 years ago, which implies that wrestling is stuck in the past and resistant towards the implementation of new knowledge and research findings in training. Accordingly, training routines and plans may be suboptimal, especially regarding the high training volume experienced by a few wrestlers.

The injured professional wrestlers felt that, in wrestling culture, ‘you are only what you perform on the mat’, and so being injured made them unworthy of interest and time from the trainer, perhaps not even being seen as human beings, making them shift from the highest peak of performance to worthlessness. Some of the included subjects would have liked an opportunity for referral to psychological support and wanted more individually based support from trainers and the federation while injured.

#### No support from the federation or trainer

**Figure 3:**
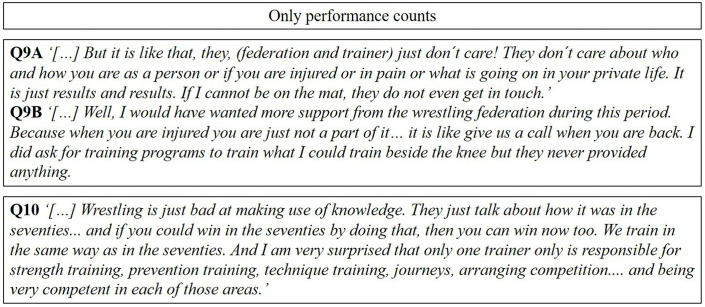
A selection of representative quotes relating to the subcategories in the third main category.

Some professional wrestlers experienced no support from trainers or the Wrestling Federation. Injured subjects reported support as the lowest when the need was the greatest (Q9A and B, figure 3).

#### A desire for a major change in the federation

Some of the injured subjects stated that wrestling training is as it was 50 years ago, and is resilient to changes, metaphorically speaking like an old dog that cannot be taught new tricks. Professional wrestlers expressed a desire for a major change in the traditionalist wrestling culture, with training designed to prevent injuries and provide access to psychological support when needed (Q10, figure 3).

### The injury’s impact on life: a wrestling with emotions

The wrestlers experienced the ACL injury and subsequent rehabilitation as a major challenge on both the physical and the mental plane. They reported the need to fight against the stress of coming back, identity losses, frustration and shattered dreams while having knee symptoms such as pain, stiffness and knee-related uncertainty. The period following the injury was experienced as dark and demanding.

Some wrestlers felt hopeless as they saw their dream to become the best in the world fade away. Others experienced feelings of guilt for being perceived as weak when a wrestler is supposed to be strong. Family and friends were mentioned as an important source of support to get through such a demanding period. Half the injured professional wrestlers who were interviewed never returned to their former level of wrestling performance.

#### Mental challenges during rehabilitation

**Figure 4:**
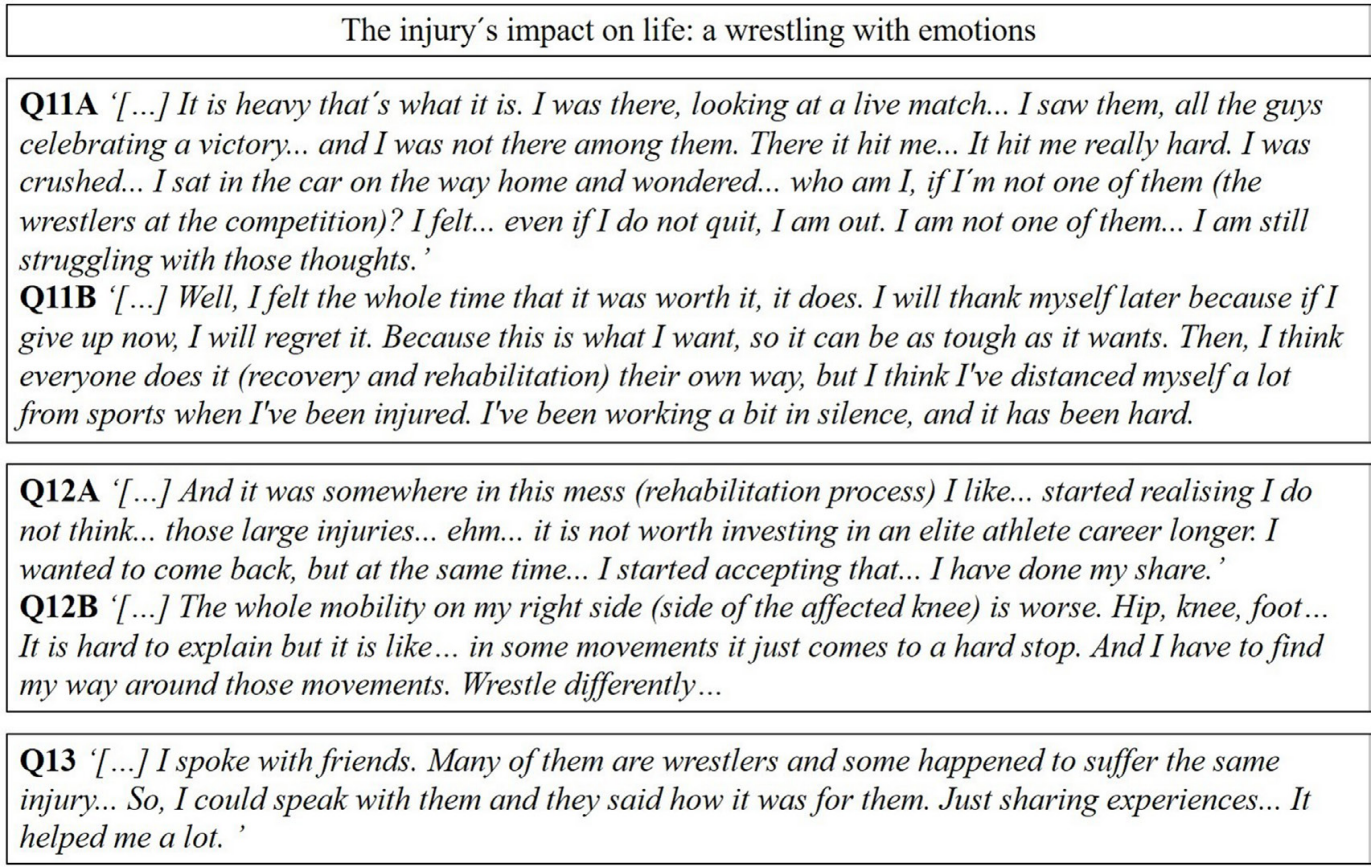
A selection of representative quotes relating to the subcategories in the fourth main category.

The included subjects experienced wrestling as a major part of their identity. Wrestling was, therefore, a very important generator of goals, ambitions and self-esteem. Suffering the ACL injury and forcing them away from the sport made the professional wrestlers question who they were and what life was without wrestling (Q11A and B, figure 4).

#### Necessary adaptations due to the injury

Several adaptations, such as a reduction in training volume, changes in wrestling style, or simply opting out of an elite career and giving more time to family, were needed due to knee-related impairments. Professional wrestlers reported long-lasting knee-related impairments, such as temporary locks, crepitation, stiffness and pain. However, most professional wrestlers said during the interview that the symptoms were manageable and that they were satisfied with having a stable knee (Q12A and B, figure 4).

#### Support from family members and friends

Perceiving support from family and friends was mentioned as very important by some wrestlers to cope with rehabilitation demands, especially in light of the loneliness perceived when they were sidelined from their wrestling club and the federation (Q 13, figure 4).

### In hindsight, personal growth

Professional wrestlers reported learning new insights from the injury and recovery process, which was seen as a process of personal growth. These new insights included thoughts of injured people and their bodies. Some wrestlers acknowledged injured people as people and not only as an injury. Other lessons mentioned were the ability to listen to their body and to find the strength to make their own decisions. However, despite the insights learnt, professional wrestlers reported regretting to have suffered an ACL injury, and, in 50% of the interviewed cases, they had to give up an elite athletic career.

#### Lessons learned

**Figure 5:**
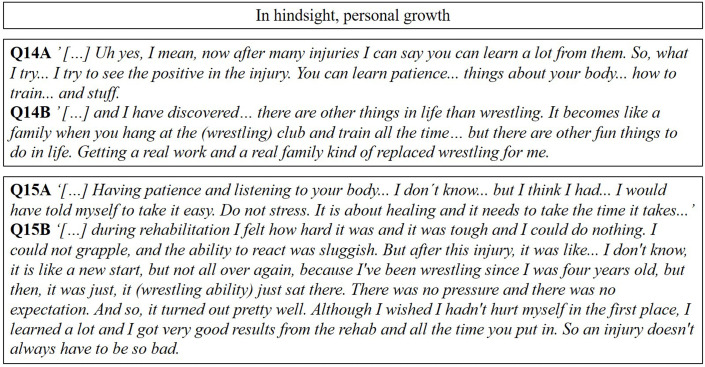
A selection of representative quotes relating to the subcategories in the fifth main category.

Some felt grateful for the understanding that life and wrestling must be pleasurable and not performed as a must. Others mentioned learning to listen to their body and acquiring new insight with regard to people who suffer ACL injuries, who need to be acknowledged as people despite being injured (Q14A and B, figure 5).

#### Alternative considerations

Professional wrestlers said they regretted not listening to their body and their feelings. The desire for a more sustainable, well-planned training schedule was common to all the included subjects. Further, wrestlers wanted not to stress through rehabilitation to be back on the mat as soon as possible but rather to allow their knee to recover fully before returning to wrestling (Q15A and B, figure 5).

## Discussion

The analysis of this interview study resulted in a single theme, divided into five main subcategories. Overall, professional wrestlers who suffered an ACL injury experienced both the injury itself and the recovery process as highly challenging. Their challenges included being unable to train as before, missing important competitions and the consequent stress, feelings of loneliness, pain and not receiving the support they desired from the federation. Rehabilitation after an ACL reconstruction has been reported as consisting of physical and psychological challenges even by non-elite athletes,^13^ and in contexts other than ACL reconstruction, patients have reported feelings of frustration and self-doubt throughout the rehabilitation process after a sports injury.^29 30^ All challenges were new, and professional wrestlers felt unprepared and unequipped to face the challenges that the ACL injury and recovery process brought. Thus, metaphorically speaking, suffering an ACL injury and recovery could be compared with facing an opponent that cannot be grappled: the desire to fight and recover was made very difficult by not knowing how to face the opponent properly. In half the cases examined in this study, the ACL injury forced the athletes to abandon their professional careers. Some reported that their injury was due to uncontrollable factors (bad luck), while others thought a long and intensive training period without adequate recovery caused their injury.

The period of recovery after ACL injury and surgery was reported as loaded with negative emotions. Negative emotions can exert a profound negative impact on the effect of treatment, as well as the recovery process following ACL reconstruction.^31^ Negative psychological response, such as fear of reinjury or lack of confidence, has been associated with poorer rehabilitation outcomes after ACL reconstruction.^32^,^34^ More specifically, when they could not perform on the mat, many professional wrestlers felt that they had been abandoned and were no longer regarded as human beings by their trainers. However, it should be mentioned that the emotion of being abandoned was perceived by professional wrestlers but perhaps not intended by trainers. The experience of feeling abandoned might have taken place despite a trainer’s best efforts to support the injured athlete. During a period of experiencing strong negative emotions, professional wrestlers considered social support from friends and family to be a key factor in being able to cope with the challenging rehabilitation period and the negative emotions associated with it. Importantly, for 50% of the included professional wrestlers, the ACL injury led to the end of their wrestling career. Social support has been shown to play a central role during ACL reconstruction rehabilitation, and it has been associated with future well-being^35^ and psychological variables associated with return to sport.^36^ Accordingly, some professional wrestlers might have ended their careers due to insufficient support during the ACL reconstruction rehabilitation. Professional wrestlers reported having learnt important lessons such as listening to their bodies and growing as a person. In a life period characterised by an event, the ACL injury, with a clear negative polarisation, professional wrestlers could see several positively charged aspects. Similar findings have been reported in patients who suffer both one or even two ACL injuries, regardless of whether they return to sport or not.^17 37^

Some professional wrestlers reported that the ACL injury happened due to bad luck, while others perceived the ACL injury to result from a long period of exhausting training. A typical situation associated with bad luck that is reported to lead to an ACL injury is when a foot is stuck on the mat, and external force (the contender) is applied to the upper body in a situation where the wrestler does not want to fall. Interestingly, the foot stuck on the mat is commonly blamed for leading to ACL injury. On the other hand, resisting falling on the back in this situation is as much to blame as the foot getting stuck on the mat. Consequently, the defence technique used where the foot gets stuck on the mat might need to be reworked to minimise ‘bad luck’ injury mechanisms. High knee abduction moments, knee abduction angles and ground reaction forces are important predictors of ACL injury.^38^ In addition, high levels of perceived stress and maladaptive responses to stress are also risk factors for injury in athletes.^39^ As a result, a long period of exhausting training might place professional wrestlers (and other professional athletes) in a situation with reduced muscular control and increased stress levels, consequently imposing a higher ACL injury risk. However, and importantly, the professional wrestlers we interviewed might have wanted to find a scapegoat to blame for the occurrence of their injury, influenced by recall and confirmation bias. Even if several of the included professional wrestlers reported that the ACL injury resulted from the high training load without recovery, this does not infer a causal relationship, and the information must be considered cautiously. Future detailed studies of the relationship between training loads and the risk of ACL injury are warranted.^40^

Healthcare professionals working with professional wrestlers should acknowledge that rehabilitation after ACL reconstruction is physically and mentally demanding. Consequently, trainers and healthcare professionals working with professional wrestlers must work actively to help wrestlers minimise the negative psychological response after an ACL reconstruction and provide every possible support. As the psychological response to any situation is highly subjective, minimising the negative psychological response should be adapted to every wrestler and situation by clear cooperation between the wrestler, trainer and other healthcare professionals involved in the rehabilitation. Trainers and healthcare professionals eventually involved in the rehabilitation need to ensure that the appropriate social support is present. However, support can be perceived differently by every unique injured athlete, and healthcare professionals need to check the athlete’s perceptions and listen to those before just trying to overwhelm the injured athlete with what the rehabilitation professional defines as ‘support’.^41^ Trainers and healthcare professionals involved in the rehabilitation of professional wrestlers can use this knowledge to pass on the message that ‘this too shall pass’, and despite all the negative emotions perceived during rehabilitation, some positive lessons can be learnt.

### Method discussion/limitations

Within content analysis, trustworthiness is a central aspect.^27 28^ Trustworthiness is divided into three core concepts: credibility, dependability and transferability. To establish credibility, the researchers must describe the research participants accurately. The respondents could only be described at the group level to ensure confidentiality. On the other hand, the researchers involved are described following the COREQ domains. Authors preconceptions were thoroughly discussed at weekly meetings to minimise preconceptions' potential effect.

Dependability refers to the certainty with which the analytical process has been carried out and the stability of the data over time. The interview guide was worked on before the study started and not changed afterwards to ensure dependability.

Transferability is the potential for extrapolating the results to other groups or situations. Transferability does not necessarily need to be an aim for a qualitative project, and the authors did not plan for the potential to transfer results. Instead, authors presented how Swedish professional wrestlers experience suffering an ACL injury and its aftermath. Consequently, the transferability of our results should be viewed with caution.

One important limitation in this study might be potential recall bias, especially since 1–240 months had elapsed between the time points for ACL reconstruction and the interview. Consequently, some professional wrestlers’ experiences and memories might be flawed by recall bias. However, happenings associated with strong positive emotions are more prone to recall bias, that is, to be reported as exaggerated. On the other hand, happenings associated with strong negative emotions tend to be remembered much more clearly.^42^ An ACL injury is perceived as a happening associated with strong negative emotions; thus, recall bias might not have influenced participants to a high degree. Nevertheless, the results should be interpreted with caution.

A further limitation could be the inclusion of only 10 professional wrestlers. The number of interviews adequate to have enough data has long been debated within qualitative studies.^43^ A frequent answer is that ‘it depends’.^43^ Factors such as, but not limited to, heterogeneity of the sample, the aim of the study, the amount of useful information obtained from each participant and the nature of the study contribute to the number of necessary interviews. Despite the number, one core aspect of qualitative research is when the data collection process no longer offers new or relevant data, sometimes called ‘saturation’. In our study, every interview was transcribed and analysed before the next interview was performed, and additional interviews were carried out until no further subcategories emerged. Consequently, we believe that we have captured representative perceptions of factors leading to a serious knee injury and the experience of an injury of this kind by professional Swedish wrestlers.

Lastly, we collected our data via ZOOM, which is reliable and is in some ways preferable to face-to-face interviews for ease of use, cost-effectiveness, management of data and security.^44^ On one hand, digital interviews allow the participants to choose a location where they feel comfortable and at ease,^45^ while, on the other hand, problems with the technology itself, participants’ lack of expertise with this technology, and loss of intimacy have been described.^44 46^ Finally, it remains unclear whether and, if so, how the use of Zoom for collecting data impacts the information obtained.

## Summary

Professional wrestlers who experienced an ACL injury found that not only the injury itself but also the subsequent recovery posed major challenges that they did not know how to deal with and that, in some cases, actually ended their professional careers. Professional wrestlers attributed their ACL injury to bad luck or large training loads and wanted more support from the wrestling community when injured. Rehabilitation professionals working with professional wrestlers must be supportive, gain wrestling-specific knowledge and provide wrestlers with tools to face this great challenge.

## Data Availability

Data are available from the corresponding author upon reasonable request.
